# Self Assembled Films of Porphyrins with Amine Groups at Different Positions: Influence of Their Orientation on the Corrosion Inhibition and the Electrocatalytic Activity

**DOI:** 10.3390/molecules17077824

**Published:** 2012-06-26

**Authors:** Koodlur Sannegowda Lokesh, Michel De Keersmaecker, Annemie Adriaens

**Affiliations:** Department of Analytical Chemistry, Ghent University, Krijgslaan 281-S12, 9000 Ghent, Belgium; Email: lokeshsk@gmail.com (K.S.L.); michel.dekeersmaecker@ugent.be (M.D.K.)

**Keywords:** porphyrin, self assembly, electrocatalysis, orientation

## Abstract

Self-assembled molecular films of two cobalt porphyrins with amine groups at different positions—(5,10,15,20-tetrakis-(2-aminophenyl) porphyrin-cobalt(II), [Co(II) (T(o-NH_2_)PP)] and (5,10,15,20-tetrakis-(4-aminophenyl) porphyrin-cobalt(II), [Co(II)(T(p-NH_2_)PP)]—were formed on a gold substrate. The functionalized surfaces were characterized using Raman spectroscopy, atomic force microscopy and electrochemical methods. Both modified gold surfaces completely mask the charge transfer of a [Fe(CN)_6_]^3−/4−^ redox couple in solution, indicating the layer is highly resistive in behavior. Electrochemical impedance spectroscopy analyses revealed that the porphyrin film with amine groups at *ortho* positions shows a higher charge-transfer resistance with a better protective behavior compared to the *para* position modified surface. Raman, AFM and EIS data suggests that an *ortho* amine positioned molecule forms a more compact layer compared to the *para*-positioned molecule. This can be explained in terms of their orientation on the gold surface. [Co(II)(T(o-NH_2_)PP)] adopted a saddle shape orientation whereas [Co(II)(T(p-NH_2_)PP)] adopted a flat orientation on the gold surface. The porphyrin modified gold electrode catalyzes the oxygen reduction at lower potentials compared to the bare gold electrode. The shift in the overvoltage was higher in case of molecules with flat orientation compared to the saddle shaped oriented porphyrin molecules on the surface.

## 1. Introduction

Porphyrin complexes with metal ions are found to be versatile model compounds for biological electron transport and for metalloenzymes. They also find numerous applications as ligands for the spectrophotometric determination of cations, stationary phases in high-pressure liquid chromatography (HPLC), biosensors, catalysis, photovoltaic cells and membrane components for ion selective electrodes, *etc.* [1,2,3,4,5,6,7]. As a result, they have been studied extensively and in particular, the redox process involving the oxidation and reduction of the central metal has been elucidated to a large extent [8,9,10,11].

Many of the applications essentially require the use of porphyrins as well packed, ordered and oriented thin films obtained from self-assembly by chemisorption [12,13,14,15,16,17], Langmuir-Blodgett deposition [18,19,20], spin coating [18,21,22], vapor-phase deposition [23,24,25] and physical vapor deposition [26,27]. These self-assembled films offer a unique strategy for constructing stable, well-defined structures on electrodes with controlled chemical features, and optimize the complexation or other supramolecular interactions at the electrode-solution interface compared to other methods. The molecular dimension of these films protects the surface by avoiding slow diffusion of electroactive species towards the electrode surface and reduces the undesirable accumulation of species on or close to the electrode surface, which may lead to electrode fouling. The introduction of a large center such as the porphyrin molecule can affect the packing preference and the organization on the electrode surface [28,29], which helps in electrocatalysis, sensing, corrosion inhibition and device applications. Additionally, fast electron transfer, good selectivity and high sensitivity can be easily achieved at the functionalized electrode [30,31].

The orientation of porphyrin molecules on the substrate depends on the length of the spacer between the ring and the gold surface. Porter and co-workers, for instance, have reported on the orientation of two different porphyrin molecules onto a gold substrate by having one or eight linker legs [32]. These studies were done with two different porphyrins with different numbers of peripheral spacers. The synthesis of trichlorosilyl, thiol and thioether derivatives of porphyrins is difficult and low yielding, and the product is highly unstable under ambient conditions. This has prompted interest towards the self-assembly of amine-substituted porphyrin molecules on metal surfaces which also make a covalent bond with the gold surface similar to that of organosulfur compounds [33,34]. Despite the promise of these chemically modified electrodes, some important issues remain unresolved, such as low coverage and orientation effect on chemical properties. It is important to understand the effect of the position of the functional groups on their packing preference, organization and orientation. Our approach towards addressing this problem involved designing a system in which it would be possible to control the spatial orientation and the coverage of the adsorbate on the surface. Important criteria for this system include a strong binding of the adsorbate and a good structural definition. In this manner, we hope to be able to use chemical manipulation to fabricate metalloporphyrin-coated electrodes with controllable properties and surface architectures, and to examine how these factors influence the protecting behavior of the gold surface and their electrochemical activity. Substrate-dependent orientation and the position-dependent orientation have been reported for phthalocyanines [35,36,37]. In order to have improved and better catalytic activity, it is essential to control the formation of the porphyrin layer at the molecular level. The immobilization of metalloporphyrins at the electrode surface for the electrocatalysis of dioxygen reduction has received significant attention [38,39].

The aim of the present study was to determine whether amino-substituted tetraphenyl porphyrins with the amine groups at two different positions: 5,10,15,20-tetrakis-(2-aminophenyl)-porphyrin-cobalt(II) [Co(II)(T(o-NH_2_)PP)] and 5,10,15,20-tetrakis-(4-aminophenyl)-porphyrin-cobalt(II) [Co(II) (T(p-NH_2_)PP)] adopt different orientations on a gold substrate. Electrochemical impedance spectroscopy was used to study the protective behavior of these films on gold surface. In addition, a comparison has been made regarding the electrocatalytic activity of the two molecules.

## 2. Results and Discussion

### 2.1. Structure of the Porphyrins

**Figure 1:**
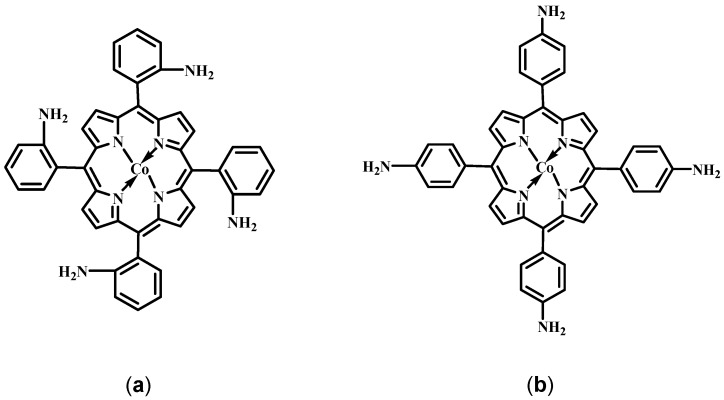
Structure of the porphyrin systems: (**a**) 5,10,15,20-tetrakis-(2-aminophenyl) porphyrin-cobalt(II), [Co(II)(T(o-NH_2_)PP)] and (**b**) 5,10,15,20-tetrakis-(4-aminophenyl) porphyrin-cobalt(II), [Co(II)(T(p-NH_2_)PP)].

The structures of [Co(II)(T(o-NH_2_)PP)] and [Co(II)(T(p-NH_2_)PP)] are shown in Figure 1. The first molecule has four amine groups at the *ortho*-position of the benzene rings and the second molecule at the *para*-position. The amine groups as well as the aromatic π-electrons in the porphyrin ring are expected to interact with the gold surface to form a stable film. The stable film formation can also be attributed to the overlap between the d-orbital of the Co(II)-ion and the gold surface [40,41,42].

### 2.2. Characterization of the Modified Gold Surfaces

**Figure 2:**
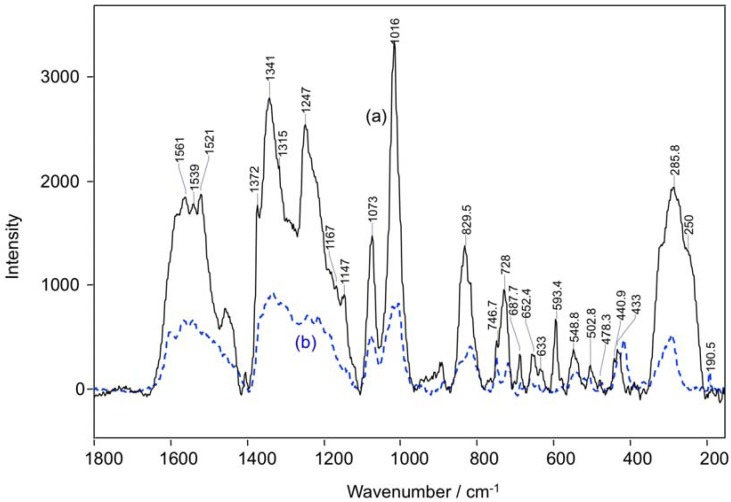
Raman spectra of a Co(II)(T(o-NH_2_)PP) (**a**) and Co(II)(T(p-NH_2_)PP) (**b**) film on a gold substrate.

The Raman spectra of Co(II)(T(o-NH_2_)PP) and Co(II)(T(p-NH_2_)PP) functionalized on the gold coupons are given in Figure 2. The Raman signals observed for the porphyrin layer on gold are in good agreement with the powder sample (not shown) as well as with data documented in the literature [43]. The spectra are dominated by strong in-plane stretching and breathing modes of the porphyrin macrocycle and are assigned based on the reported literature [43]. It is expected that the adsorption process leads to an interaction of the gold with the nitrogen of the peripheral NH_2_-group giving rise to a gold-nitrogen stretch [44] in addition to the strong interaction between the π-electrons of porphyrin and the gold surface, but the weak band that should appear at 230 cm^−1^ for the gold-nitrogen stretch, could not be distinguished as the porphyrin shows an intense δ(C=C) band at 250 cm^−1^.

Table 1 gives an overview of the various bands and their corresponding assignments. The stretching vibration of the pyrrole ring on the porphyrin macrocycle is observed at 1,341 cm^−1^. The macrocyclic vibration observed at 1,073 cm^−1^ can mainly be assigned to ν(C=C). The pyrrole ring out-of-plane deformation is observed at 728 cm^−1^ and the pyrrole ring breathing vibration appears at 747 cm^−1^ [34,43].

**Table 1:** Raman spectral bands of a Co(II)(T(o-NH_2_)PP) and Co(II)(T(p-NH_2_)PP) film on gold, with the respective assignments [43,44].

Raman shift, cm^−1^	Assignments [ν, Stretching; δ, Bending]
1561	ν (C_β_C_β_)
1540	ν (C_α_C_m_)_sym_
1372	ν (C_α_C_β_), ν (CβCs), ν (pyr half-ring)_sym_
1341	ν (pyr half-ring)_sym_
1247	δ (C_m_H)
1073	ν (C_β_C_β_)_asym_
1016	ν (C_β_C_β_)_asym_
830	ν (pyr breathing)
747	ν (pyr breathing)
728	δ (pyr def)sym
688	δ (pyr def)sym
593	δ (pyr def)asym
549	δ (pyr rot)
250	δ (C_β_C_β_)_sym_

**Figure 3:**
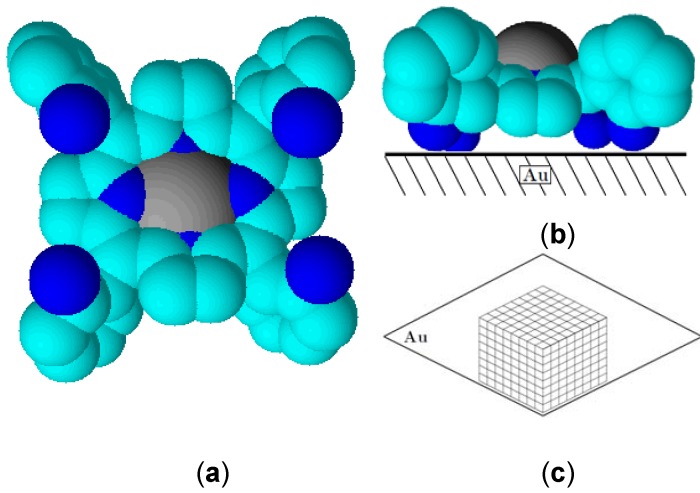
Space-filling models showing top view (**a**) and side view (**b**) of the Co (II)(T(o-NH_2_)PP) adsorbed on the gold electrode. Also shown is the formation of the porphyrin multilayer (**c**).

**Figure 4:**
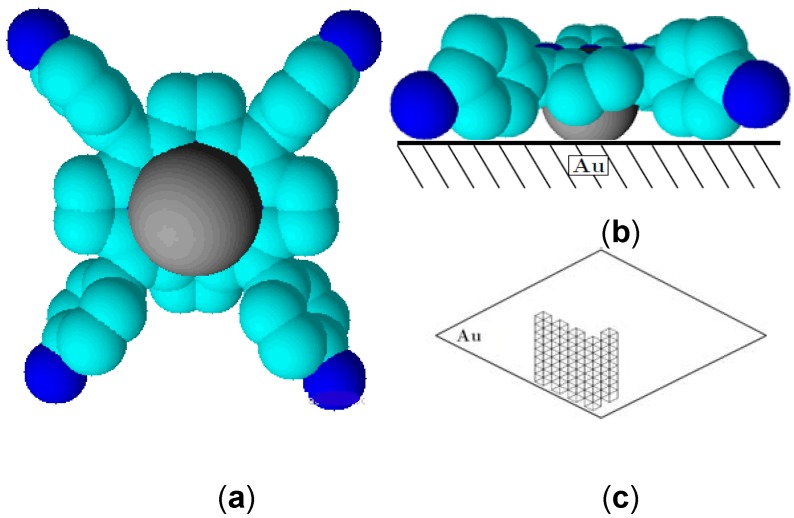
Space-filling models showing top view (**a**) and side view (**b**) of the Co(II)(T(p-NH_2_)PP) adsorbed on the gold electrode. Also shown is the formation of the porphyrin multilayer (**c**).

Although both molecules form an assembly, we observe that the intensity of the Raman peaks are higher for Co(II)(T(o-NH_2_)PP) compared to Co(II)(T(p-NH_2_)PP), indicating the amount of Co(II)(T(o-NH_2_)PP) adsorbed on the surface is comparatively higher than that of Co(II)(T(p-NH_2_)PP). This can be understood by the difference in the orientation of the two porphyrin molecules under study occupies on a gold surface [35,36]. Figure 3a,b and Figure 4a,b show the three-dimensional orientation of both porphyrin molecules on the gold surface based on the ortho and para position of the amine substituent. It is believed that both porphyrin molecules occupy a flat orientation on the gold surface, but in case of the Co(II)(T(p-NH_2_)PP) molecule, some of the amine-substituents are turned outwards (Figure 3b and Figure 4b). Figure 3c and Figure 4c propose a diagram of how the formation of the multilayer should proceed based on the 3-dimensional orientation of the porphyrin complexes. The multilayer of the Co(II)(T(o-NH_2_)PP) layer can be stacked closely to each other because of its saddle shape conformation shown in Figure 3b. The Co(II)(T(o-NH_2_)PP) layer, however, cannot be stacked that way because of the amine groups which point outwards and leave a gap between the molecules due to repulsion interactions. The higher signal to noise ratio observed for the multilayer film is due to the larger Raman cross-section of porphyrin molecules and the surface enhancement effect of the Raman signal [45]. 

**Figure 5:**
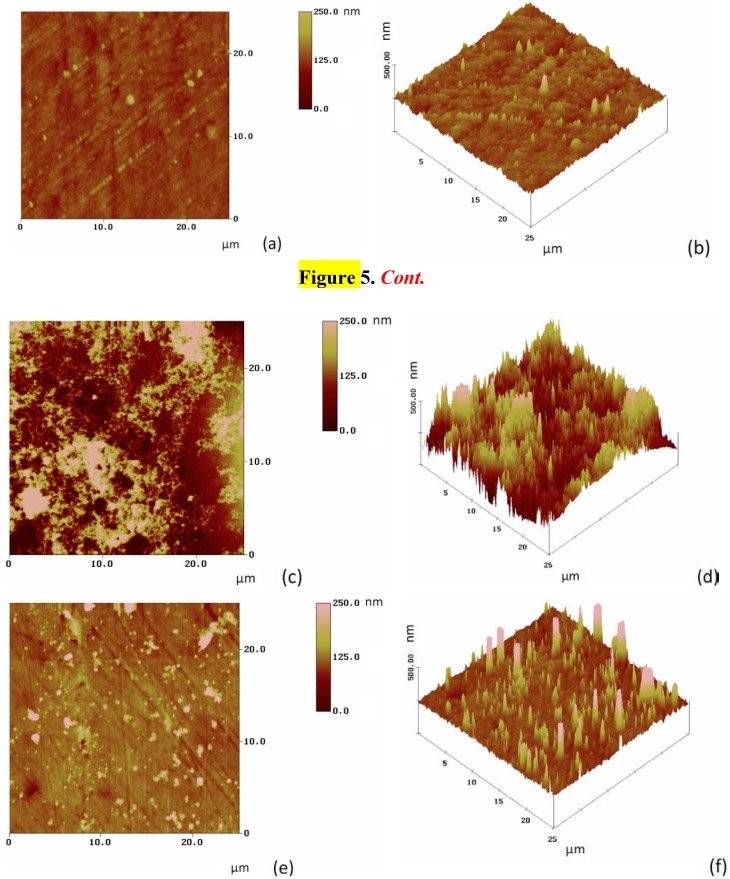
AFM images showing top view (contour plot) and 3D surface plot respectively of unmodified gold surface (**a** and **b**), Co(II)(T(o-NH_2_)PP) modified gold surface (**c** and **d**) and Co(II)(T(p-NH_2_)PP) modified gold surface (**e** and **f**).

AFM experiments were performed to study the surface morphology of the porphyrin layers deposited on the gold surface (Figure 5). The top view and the 3-dimensional surface plot of the unmodified gold surface are shown in Figure 5a,b, respectively. The visible scratches on the bare gold surface in Figure 5a are a consequence of the polishing with SiC and alumina.

Changes in surface morphology were quantified using root-mean-square (RMS) roughness values (R_q_). The value increases from 15 nm for the bare gold sample to 79 and 37 nm for the Co(II)(T(o-NH_2_)PP) and Co(II)(T(p-NH_2_)PP) modified gold surfaces respectively. The larger RMS value means an increase in surface roughness after the deposition of the two porphyrin layers on the gold surfaces, which can be clearly deduced from the 3D surface plots (Figure 5d,f *vs.* Figure 5b). In addition, the top and 3D views clearly show the variation in the coverage and the morphology between the two deposited films: while the Co(II)(T(o-NH_2_)PP) modified surface is completely and almost uniformly covered (Figure 5c), where as the Co(II)(T(p-NH_2_)PP) modified surface is partly covered with a few islands of porphyrin on the surface (Figure 5e).

**Figure 6:**
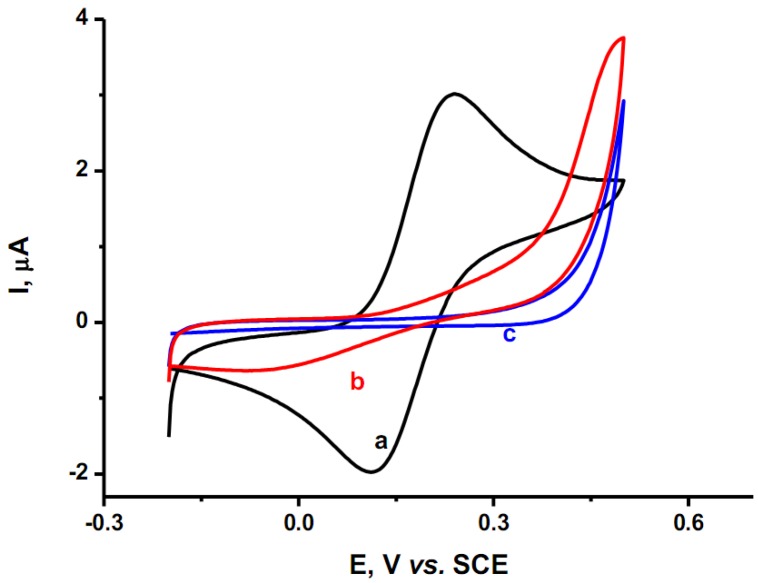
Cyclic voltammograms in a phosphate buffer of pH 7.0 with 1 mM K_4_Fe(CN)_6_ for the bare gold electrode (**a**) and the Co(II)(T(o-NH_2_)PP) (**b**) and Co(II)(T(p-NH_2_)PP) (**c**) modified gold electrodes at a scan rate of 50 mV/s.

Cyclic voltammograms recorded in a 1 mM potassium ferrocyanide solution with phosphate buffer on a bare gold and the two modified gold surfaces are shown in Figure 6. The voltammogram of the bare gold electrode clearly shows the oxidation and reduction peaks of the ferri/ferrocyanide couple at a potential of 0.23 and 0.12 V *vs.* SCE (curve a) according to the following reaction [46].



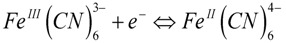



After modifying the gold electrode with either Co(II)(T(o-NH_2_)PP) or Co(II)(T(p-NH_2_)PP), the cyclic voltammogram no longer shows any redox peaks (curves b and c). Their suppression is consistent with the presence of a film layer or coating on the gold electrodes [47].

The porphyrin-modified electrodes completely mask the charge transfer from electrode to electrolyte and are highly resistive in nature. At higher oxidative potentials, the bare gold electrode shows high currents due to the formation of gold oxides on the surface as a result of the presence of some dissolved oxygen gas. At the porphyrin-modified electrodes, the gold oxidation reactions are suppressed due to the presence of a strong isolating barrier. However at a potential of 0.5 V, a large oxidation peak is seen and it is possible that gold oxides are formed under the layer and therefore the barrier is broken and also the Fe(CN)_6_^4−^ present is oxidized. Another possibility could be that at slightly higher potentials, electrons are transported along the coating from the electrode to the solution indicating a not fully isolating barrier.

**Figure 7:**
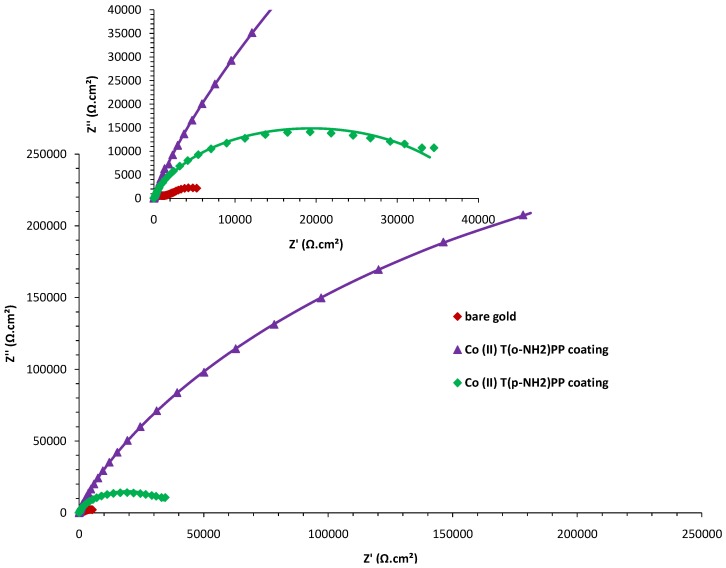
Nyquist plot: the experimental data points are fitted by the solid lines. Inset: magnification of the Nyquist plot in the high frequency region.

Electrochemical impedance spectroscopy (EIS) measurements were used to study the kinetics and diffusion characteristics at the porphyrin-gold interface. The impedance measurements were carried out in a phosphate buffer with 1 mM potassium ferrocyanide at a potential of 0.12 V. The Nyquist plots (Z_imaginary_ *vs*. Z_real_) of the bare and both modified gold electrodes are shown in Figure 7.

They exhibit a characteristic semicircle at high frequencies and a straight line at low frequencies, corresponding respectively to kinetic and diffusion processes at the gold electrode. In case of the Co(II)(T(o-NH_2_)PP) modified sample, only the semicircle is seen, which indicates a decrease in diffusion of compounds through the layer because of a well-formed porphyrin film [48]. The slightly depressed nature of the semicircles in the Nyquist plots, even for the bare gold electrode, indicates so-called impedance dispersion. The latter can be explained by a number of factors such as the roughness of the electrode surface, varying thickness of the film, non-ideal behavior of the coating and non-uniform distribution of the current density of the surface [48,49].

**Figure 8:**
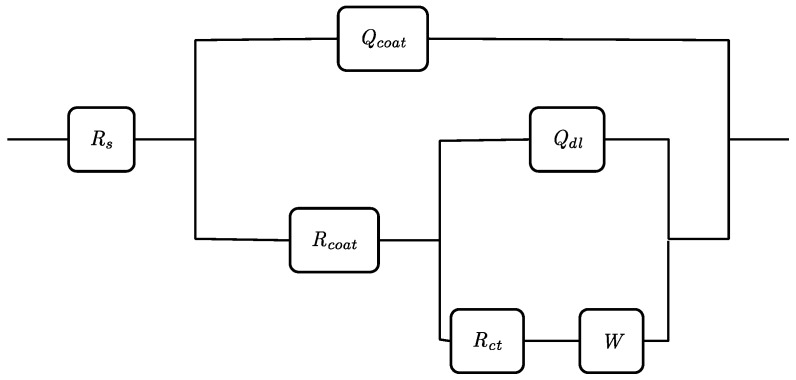
Electric circuit used to simulate the experimentally recorded electrochemical impedance spectra.

To fit the EIS data, the spectra were modeled using an equivalent electric circuit using mixed kinetic and diffusion control, taking into account the number of time constants deduced from the phase plot. Figure 8 shows an electric circuit with two time constants where R_s_ is the resistance of the electrolyte, R_coat_ the pore resistance of the film and Q_coat_ the constant phase element representing the capacitance due to the coating barrier. In parallel with R_coat_, we have Q_dl_ the constant phase element representing the double layer, R_ct_ the electron-transfer resistance representing the most appropriate parameter to monitor the protective properties of the film and W the Warburg impedance [50].

The estimated parameters used to fit the impedance spectra in Figure 7 are presented in Table 2. It is clear that the double layer capacitance values, C_dl_, are much smaller for the coated samples than that for the bare one (ca. factor of 40).

**Table 2:** Summary of the estimated electrochemical impedance parameters obtained for the electrodes at the measured OCP using the electric circuit in Figure 8 (The CPE values are calculated back to approximate capacitance values).

	R_s_ (Ωcm^2^)	C_coat_ (µFcm^−2^)	R_coat_ (Ωcm^−2^)	C_dl_ (µFcm^−2^)	R_ct_ (.10^3^ Ωcm^2^)	W (.10^−3^ Ω^−1^cm^−2^ s^−1/2^)
**bare gold**	6.65	38.11	1262.5	925.37	7.32	10.69
**Co(II) (T(p-NH_2_)PP)**	6.61	28.75	513.4	24.53	844.20	7.96
**Co(II) (T(o-NH_2_)PP)**	6.70	12.58	295.5	15.82	3002.94	4.36

The double layer capacitance can be considered as a measure of the area over which no coating has been deposited. The relative area is calculated as follows [48]:


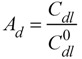



where C^0^_dl_ is the area specific double layer capacitance of the uncoated metal. Results for the Co(II)(T(p-NH_2_)PP) and Co(II)(T(o-NH_2_)PP) covered electrodes give 0.03 and 0.015, respectively, which indicates a bigger inhibited surface with Co(II) (T(o-NH_2_)PP).

Also the R_ct_ values of both porphyrin coated gold electrodes are higher compared to the bare gold electrode and depend on the type of porphyrin. The higher R_ct_ value observed for Co(II)(T(o-NH_2_)PP) compared to Co(II)(T(p-NH_2_)PP) is due to a more uniform and compact packing. The data also reveal that the R_ct_ and C_dl_ values vary in an inverse proportion, which can also be attributed to a protective layer [51].

Following this reasoning, we can conclude the Co (II)(T(o-NH_2_)PP) forms the most corrosion-resistant layer. The inhibition efficiency can be subsequently calculated from the impedance parameters using the following relationship [52]: 



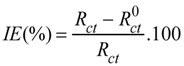



where R_ct_ and R^0^_ct_ are the charge transfer resistances in the presence and absence of the porphyrin inhibitors, respectively. The inhibition efficiencies, 99.75% and 99.13%, calculated respectively for the Co(II)(T(o-NH_2_)PP) and Co(II)(T(p-NH_2_)PP) coatings suggest in both cases that the layer protects the gold electrode from electron transfer with the bulk solution. 

**Figure 9:**
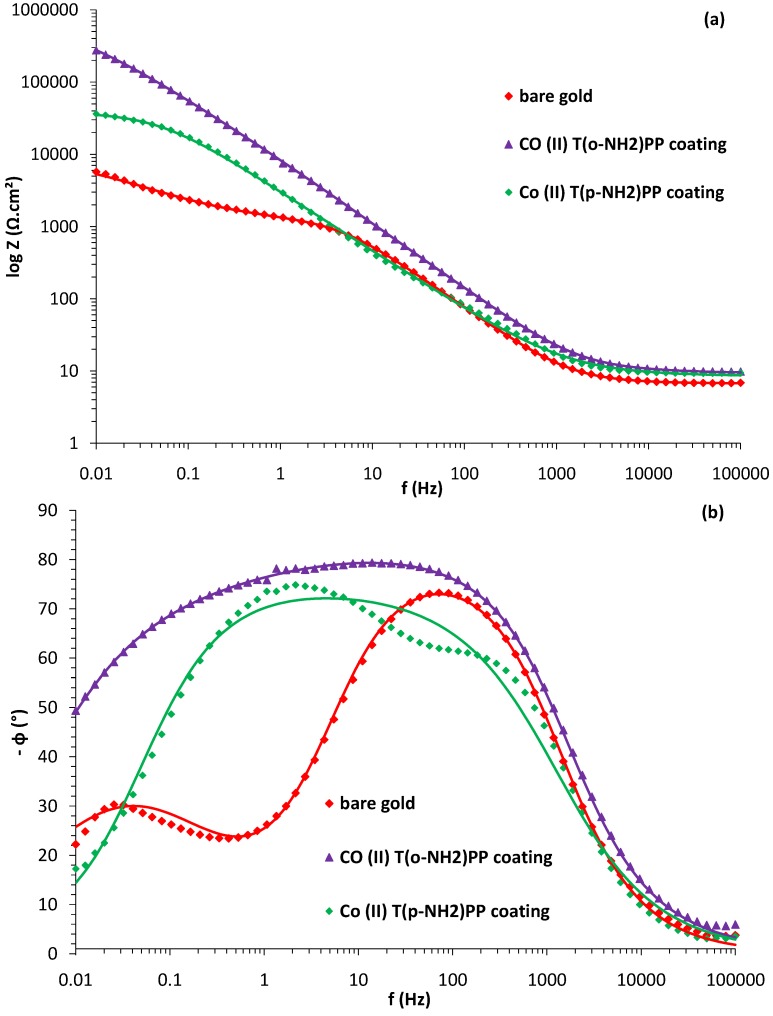
(**a**) log |Z| *vs.* (f) plot; (**b**) Bode phase plot of the Co(II)(T(o-NH_2_)PP) and Co(II)(T(p-NH_2_)PP) modified gold electrode in a phosphate buffer of pH 7.0 containing 1 mM K_4_Fe(CN)_6_. The reduction potential of Fe^3+^/Fe^4+^ = 0.12V (*vs.* SCE).

Comparing the impedance *vs*. frequency plots (Figure 9a), one can conclude that the impedance points for the treated surfaces are higher at each frequency compared to the bare gold surface. The plot for the bare gold surface demonstrates a plateau, which implies easy electron transfer. The porphyrin modified gold electrodes, on the other hand, show a straight line without independent-frequency plateau, which means the electrolyte can penetrate the coating, but no electron transfer process takes place at the gold/coating interface [52]. Grandle *et al.* [53] observed that the maximum impedance at low frequency, Z_max_, is the most useful and reliable parameter to evaluate coatings. Based on this, we can conclude that the Co(II)(T(o-NH_2_)PP) provides the best protective layer.

The phase angle *vs*. frequency plot (Figure 9b) of the bare gold sample shows two loops in the capacitive quadrant and therefore two well-defined time constants are attributed to the charge transfer process and to the diffusion of the electrolyte through the corrosion layer [54]. On the other hand, plots of both modified samples show a very broad phase distribution (only one time constant), which indicates that the complete surface is protected by the porphyrin coating due to phase values closer to a pure capacitance. The high frequency time constant remains the same compared to the bare gold, but shows a higher phase angle value in the capacitive quadrant.

### 2.4. Explanation of the Porphyrin Film Formation

The interaction between Co(II)(T(o-NH_2_)PP) and the gold surface can be explained based on two types of interactions [55]. The interaction between Co(II)(o-NH_2_)PP) and the gold surface is based on an interaction between the cobalt ion and the gold surface and between the amine phenyl substituents and the surface. The amine phenyl substituents turned inside due to *ortho*-position on the phenyl ring provides a twist angle of the phenyl substituents of roughly 60° relative to the porphyrin plane, which was consistently reported in several publications as can be seen in Figure 3a,b [56,57,58]. The well-protective properties of this porphyrin coating results from this saddle shape conformation and tomography. These initial formed structures on the gold surface provide an ideal base for the self-assembly in ordered domains close together based on intermolecular π-π or T-type interactions between the phenyl and the porphyrin rings as shown in Figure 3c. This way multilayer structures can be formed, which can form an electron transfer barrier on the gold surface. The interaction between Co(II)(T(p-NH_2_)PP) and the gold surface, in Figure 4a,b, is also based on the same interactions, which means the porphyrin molecule interacts with the gold surface and stays completely flat. A big difference, however, is that with an amine-group on the *para*-position, the group turns away from the porphyrin ring, which means the intermolecular interactions form clear islands on the electrode as can be seen in the AFM picture (Figure 5e,f and Figure 4c). Due to the steric hindrance (and repulsive behavior) of the out-turned amine groups of the porphyrin molecule bound on the surface, no other molecule can interact with the surface closely next to it. This means a small cavity will exist between the different porphyrin molecules bound on the gold surface. The multilayer will be formed on top of each porphyrin structure bound to the gold surface, which will lead to lower electron transfer resistance (Figure 4c).

### 2.5. Electrocatalytic Activity

**Figure 10:**
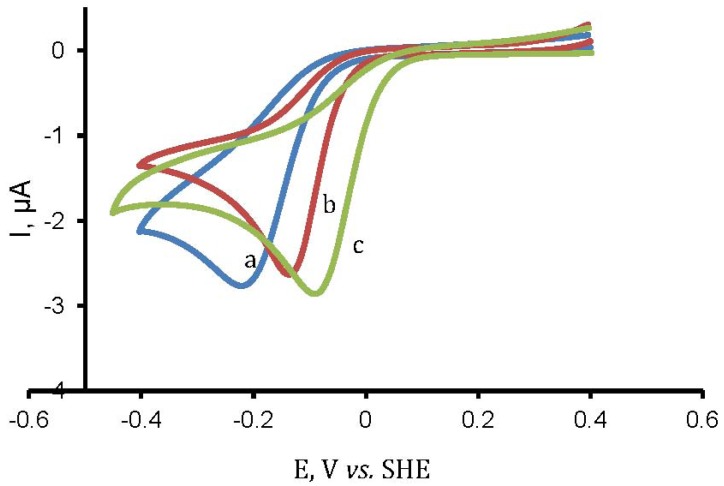
Cyclic voltammetric response for oxygen reduction on (**a**) a bare gold electrode and (**b**) Co(II)(T(o-NH_2_)PP) and (**c**) Co(II)(T(p-NH_2_)PP) modified gold electrode at 50 mV/s in phosphate buffer (pH =7.0).

Figure 10 shows the cyclic voltammograms obtained for the bare gold electrode and the Co(II)(T(o-NH_2_)PP) and Co(II)(T(p-NH_2_)PP) modified gold electrodes in a phosphate buffer (pH = 7.0) at a scan rate of 50 mV/s. The reduction peak of oxygen shows a pronounced shift towards a more oxidative potential for the porphyrin modified gold electrodes compared to the bare gold electrode. 

This behavior indicates that the porphyrin modified gold electrodes act as a mediator to shuttle electrons between oxygen in the bulk electrolyte and the gold working electrode and facilitate the electrochemical regeneration following the electron exchange with oxygen. The shift in the overvoltage has also been reported for the catalysis of other small molecules with porphyrin modified electrodes [59]. The catalytic activity can be attributed to the reduction of the central metal ion cobalt. The variation of the shift in the overvoltage obtained for the reduction of oxygen in case of Co(II)(T(o-NH_2_)PP) is 95 mV and Co(II)(T(p-NH_2_)PP) is 140 mV, can be explained by the different orientations they adopt on the gold surface. Since Co(II)(T(o-NH_2_)PP) adopts an almost perpendicular orientation on the gold surface, it is difficult for all the Co(II) ions to take part in the catalysis of oxygen [35]. However in case of Co(II)(T(p-NH_2_)PP), the metal center is well exposed to the outer environment as it adopts a flat orientation on the gold surface and it helps all the cobalt ions to take part in the catalysis of oxygen. Hence, we observe a higher shift in the overvoltage for Co(II)(T(p-NH_2_)PP) compared to Co(II)(T(o-NH_2_)PP) for the reduction of oxygen even though surface concentration of Co(II)(T(p-NH_2_)PP) is comparatively less than Co(II)(T(o-NH_2_)PP) as observed from AFM, Raman and electrochemical data. This observation clearly confirms that for electrocatalysis, the suitable orientation of the molecules on the surface determines the electron transferability and not the amount of the porphyrin molecule present on the surface.

## 3. Experimental

### 3.1. Materials

Co(II)(T(o-NH_2_)PP) and Co(II)(T(p-NH_2_)PP) were purchased from Porphyrin Systems (Appen, Germany) and used as such without any purification. Dimethyl sulfoxide (DMSO, 98%, Sigma-Aldrich, Bornem, Belgium), a phosphate buffer (pH 7.0, Sigma-Aldrich, Bornem, Belgium) and potassium ferrocyanide [K_4_Fe(CN)_6_, Merck Eurolab, Leicestershire, UK] were all of analytical grade. Inlaid gold disk electrodes with a surface area of 0.0201 cm^2^ (BASi, Warwickshire, UK) were used for the electrochemical measurements, while gold coupons of 3 mm diameter (Goodfellow, Huntingdon, UK) were used for the Raman and AFM studies. Both substrates were polished using polishing cloth (12” microcloth PSA 10/PK, Buehler, Dusseldorf, Germany) with sequentially 1.0 μm and 0.5 μm alumina/water solutions. The electrodes/coupons were consequently washed in water and later on in ethanol while being sonicated for 10 min in an ultrasonic bath.

### 3.2. Formation of a Self Assembled Film of Porphyrin on Gold

Co(II)(T(o-NH_2_)PP) and Co(II)(T(p-NH_2_)PP) were dissolved separately in DMSO (1 mM solution) in an ultrasonic bath for 1 h, after which the pre-cleaned gold samples were immersed in the dissolved porphyrin solution for a duration of 48 h. After removing the samples from the solution, they were washed thoroughly with DMSO and sequentially with ethanol and stored in a desiccator to avoid the effect of humidity and contamination of the air before use.

### 3.3. Instrumentation and Experimental Details

A confocal Raman spectrometer Senterra R200-L (Bruker, Ettlingen, Germany) was used to analyze the samples. Raman spectra were recorded using a 785 nm diode laser with a power of 300 mW at the source. An Olympus BX series microscope (Ettlingen, Germany), coupled to the spectrometer was used for the visualization of the sample and for the microanalysis with an objective lens of 50× magnification. The spectrometer is equipped with a thermo-electrically cooled CCD detector (1024 × 256 pixels). Raman spectra were recorded on a multi-laser Bruker instrument (Ettlingen, Germany) in the wave number region of 100–1,700 cm^−1^ for the film on the gold substrate. For each spectrum 15 accumulations of 30 s were recorded.

AFM images were obtained in ambient conditions with a multimode scanning probe microscope (Digital Instruments, New York, NY, USA) equipped with a Nanoscope IIIa controler. 25 μm scans were recorded in tapping mode with a silicon cantilever (OTESPA-Veeco, New York, NY, USA). The recorded images were modified with an X and Y plane fit auto procedure using Nanoscope software version 4.43r8.

Cyclic voltammetry was used to examine the charge-transfer behavior of the bare gold and both modified surfaces and also to study their electrocatalytic behavior. Experiments were performed using an Autolab potentiostat (PGSTAT 100, Utrecht, The Netherlands) with GPES software, in a three-electrode system with saturated calomel electrode (SCE) as reference, a bare gold or gold modified electrode as working electrode and a carbon counter electrode. The electrochemical experiments were performed in a phosphate buffer of pH 7.0 at a scan rate of 50 mV/s in an inert atmosphere by purging with nitrogen gas (Air Liquide Alphagaz 1 accuracies from % to 10 ppm) for 20 min before each experiment. 

Electrochemical impedance measurements were carried out using an Autolab (PGSTAT 20, Utrecht, The Netherlands) with the frequency response analysis (FRA) software, in a frequency range of 1 MHz–10 mHz with an amplitude of 5 mV in a 1 mM K_4_Fe(CN)_6_ solution in a phosphate buffer at a potential of 0.12 V to study the charge-transfer behavior of the bare and modified gold electrodes. The reduction of oxygen was studied using these modified electrodes in phosphate buffer (pH 7.0) containing dissolved oxygen at a scan rate of 50 mV/s.

## 4. Conclusions

We have successfully formed films of the amine-substituted cobalt porphyrins Co(II) (T(o-NH_2_)PP) and Co(II) (T(p-NH_2_)PP) on a gold surface and these modified surfaces were characterized by Raman, AFM and electrochemical techniques. The chemical adsorption is due to the strong interaction of peripheral amine groups on porphyrin with the gold in addition to the strong interaction between gold and porphyrin. The modified surface suppresses the charge transfer of the Fe^II+/III+^ redox couple. The charge transfer resistance is almost four times higher for Co(II)(T(o-NH_2_)PP) with an effective protecting behavior compared to Co(II)(T(p-NH_2_)PP). Raman, AFM and electrochemical results suggest that Co(II)(T(o-NH_2_)PP) is expected to adsorb with a perpendicular orientation on the gold surface, whereas Co(II)(T(p-NH_2_)PP) adopts a flat orientation on the surface. In case of Co(II)(T(o-NH_2_)PP) only one or two amine of each porphyrin attaches to gold, whereas in the case of Co(II)(T(p-NH_2_)PP), it is expected that two or more amine groups interacts with gold. Both the porphyrin modified gold electrodes found to shift the overpotential towards a more positive potential for the reduction of molecular oxygen by approximately 100 mV. The larger shift in the overpotential for Co(II)(T(p-NH_2_)PP) modified electrode for the oxygen reduction is due to the direct exposure of cobalt central metal atom to the analyte, whereas the diminished catalytic activity of Co(II)(T(o-NH_2_)PP) can be ascribed to the non-accessibility of cobalt atom to the analyte, which are well packed and perpendicular to the surface. This clearly indicates that it is the preferred and specific orientation that matters in the catalysis and not the quantity of the catalyst. The orientation and packing of the molecule on the surface finds application in selective catalysis, sensing and in the fabrication of compact and robust molecular electronic devices.
